# Effectiveness of differing levels of support for family meals on obesity prevention among head start preschoolers: the simply dinner study

**DOI:** 10.1186/s12889-017-4074-5

**Published:** 2017-02-10

**Authors:** Holly E. Brophy-Herb, Mildred Horodynski, Dawn Contreras, Jean Kerver, Niko Kaciroti, Mara Stein, Hannah Jong Lee, Brittany Motz, Sheilah Hebert, Erika Prine, Candace Gardiner, Laurie A. Van Egeren, Julie C. Lumeng

**Affiliations:** 1 Department of Human Development and Family Studies, Michigan State University, 552 West Circle Drive, 48824 East Lansing, MI USA; 2 College of Nursing, Michigan State University, 1355 Bogue Street, 48824 East Lansing, MI USA; 3 MSU Extension, Michigan State University, 446 West Circle Drive, 48824 East Lansing, MI USA; 4 Department of Epidemiology and Biostatistics, Michigan State University, 220 Trowbridge Road, 48824 East Lansing, MI USA; 5 Center for Human Growth and Development, University of Michigan, 300 North Ingalls, 48104 Ann Arbor, MI USA; 6 University Outreach and Engagement, Michigan State University, Kellogg Center, 219 South Harrison, 48824 East Lansing, MI USA; 7 Department of Pediatrics, Medical School, University of Michigan, 1500 East Medical Center Drive, 48109 Ann Arbor, MI USA; 8 Department of Nutritional Sciences, School of Public Health, University of Michigan, 1415 Washington Heights, 48109 Ann Arbor, MI USA; 9 Department of Biostatistics, School of Public Health, University of Michigan, 1415 Washington Heights, 48109 Ann Arbor, MI USA

**Keywords:** Obesity prevention, Low-income children, Meals, Preschoolers, Head start, Intervention study

## Abstract

**Background:**

Despite slight decreases in obesity prevalence in children, nearly 25% of preschool-aged children are overweight or obese. Most interventions focused on promoting family meals as an obesity-prevention strategy target meal planning skills, knowledge and modeling of healthy eating without addressing the practical resources that enable implementation of family meals. There is a striking lack of evidence about what level of resources low-income parents need to implement family meals. This study will identify resources most effective in promoting family meals and, subsequently, test associations among the frequency of family meals, dietary quality and children’s adiposity indices among children enrolled in Head Start.

**Methods:**

The Multiphase Optimization Strategy, employed in this study, is a cutting-edge approach to maximizing resources in behavioral interventions by identifying the most effective intervention components. We are currently testing the main, additive and interactive effects of 6 intervention components, thought to support family meals, on family meal frequency and dietary quality (Primary Outcomes) as compared to Usual Head Start Exposure in a Screening Phase (N = 512 low-income families). Components yielding the most robust effects will be bundled and evaluated in a two-group randomized controlled trial (intervention and Usual Head Start Exposure) in the Confirming Phase (N = 250), testing the effects of the bundled intervention on children’s adiposity indices (Primary Outcomes; body mass index and skinfolds). The current intervention components include: (1) home delivery of pre-made healthy family meals; (2) home delivery of healthy meal ingredients; (3) community kitchens in which parents make healthy meals to cook at home; (4) healthy eating classes; (5) cooking demonstrations; and (6) cookware/flatware delivery. Secondary outcomes include cooking self-efficacy and family mealtime barriers. Moderators of the intervention include family functioning and food security. Process evaluation data includes fidelity, attendance/use of supports, and satisfaction.

**Discussion:**

Results will advance fundamental science and translational research by generating new knowledge of effective intervention components more rapidly and efficiently than the standard randomized controlled trial approach evaluating a bundled intervention alone. Study results will have implications for funding decisions within public programs to implement and disseminate effective interventions to prevent obesity in children.

**Trial registration:**

Clincaltrials.gov Identifier NCT02487251; Registered June 26, 2015.

## Background

Despite slight decreases in obesity prevalence in low-income children in recent years [1], nearly 25% of children under age 5 years are overweight or obese [2]. Moreover, socioeconomic disparities in early childhood place children from low-income families at 1.5 to 2 times higher risk for obesity as compared to children from middle- to upper-income homes [3, 4]. Given that early obesity is associated with increased risk for lifelong obesity and comorbid health outcomes including cardiovascular disease and diabetes [5–7], obesity prevention targeting low-income children is a critical public health priority. Recently, obesity interventions have turned toward the promotion of family meals as an obesity prevention strategy [8–10]. While regular family meals are a key family routine relevant to obesity prevention [9, 11, 12], compared to the past, families are preparing and sharing fewer family meals [13–15] at the expense of dietary quality [16–18]. Although parents and children report enjoying and valuing family meals [19–23], they often are unable to implement them [21]. Reported barriers to family meals include limited time for meal planning and preparation [21], selecting meals and challenges with cooking [24].

While families with economic resources find planning and implementing family meals challenging [24–26], barriers to family meals among low-income families are intensified [27]. Most family mealtime intervention approaches have targeted meal planning, cooking skills and knowledge [28, 29]) and parental modeling of healthy eating [30], intervention foci that may not align with the needs of families with limited resources. Specifically, strategies to date disregard the practical resources (hereafter termed “supports”) that would enable meal planning and implementation. There is a striking lack of evidence about what level of supports low-income parents need to implement family meals. Only imparting cooking skills, nutrition knowledge, and meal planning strategies may not be enough to result in behavior change (e.g., increasing frequency of regular family meals) among low-income, stressed populations.

Our approach is built on the premises that: 1) enhancing the ability of parents to implement family meals is critical to effective obesity prevention; and 2) when parents do not implement regular family meals, it is not solely due to a lack of nutrition education or knowledge, but to the combined adverse effects of poverty on the resources available to parents to plan and execute family meals. A crucial step in the future development of obesity prevention efforts is identifying what supports are needed to aid low-income families in planning and implementing family meals. Without such information, effective interventions cannot be planned. We propose that the provision of concrete supports is also necessary to promote family meals, increase dietary quality and improve children’s adiposity indices in a low-income population. Thus, our study is designed to test the most extreme levels of support for the implementation of family meals. We are testing 6 intervention components (fully described in \), ranging from the most to least intense forms of support, in the Screening Phase of the study, scheduled to be completed in December 2017: (1) Meal Delivery- MD: home delivery of pre-made healthy family meals including recipes that are ready to heat and eat; (2) Ingredient Delivery- ID: home delivery of ingredients with recipes to make and cook healthy family meals; (3) Community Kitchen- CK: sessions in which families make healthy meals with recipes to take home and cook; (4) Didactics Healthy Eating classes with recipes via the Parents of Preschoolers- POPS curriculum; (5) Cooking Demonstration- CD: demonstration of meal preparation with recipes; and (6) Cookware/Flatware: delivery of flatware/ cookware to utilize for family meals. Using the innovative Multiphase Optimization Strategy (MOST [31, 32];), a cutting-edge approach to maximizing resources in behavioral interventions by identifying the most robust and efficient intervention model possible, we are first testing the main, additive and interactive effects of these 6 practical supports for healthy family meals in a Screening Phrase, identifying the intervention components most robustly associated with increased family meals and improvements in dietary quality. Next, the identified intervention components will be bundled and the effects of the “final” intervention on children’s adiposity indices will be evaluated via a randomized controlled trial (RCT) in the Confirming Phase of the study set to begin in September 2018.

### Study aims and hypotheses

The aims of the Simply Dinner (SD) study are to (1) identify SD intervention components, and combinations of components, most robustly associated with the frequency of family meals and dietary quality at home over eight weeks, and (2) to test the identified SD activities in a randomized controlled trial to examine their effects on children’s adiposity indices over the school year in a sample of children enrolled in Head Start. In the Screening Phase we hypothesize that cumulative SD components will be related to increases in the frequency of family meals at home and improvements in children’s dietary quality, the two primary outcomes of the Screening Phase. In the Confirming Phase, children’s adiposity indices, body mass index and triceps skinfolds are the primary outcomes.

**Fig. 1:**
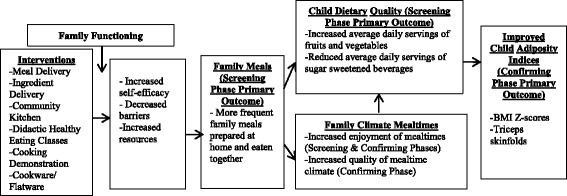
Conceptual Model

The processes through which we expect changes in children’s adiposity indices will occur are as follows and are illustrated in our conceptual model (Fig. 1). We hypothesize that parents’ increased self-efficacy, decreased barriers (time/effort) to meals, and greater resources to plan and implement healthy meals will mediate the effects of the interventions on the frequency of family meals. Moreover, improvement in children’s dietary quality is expected to mediate the effects of more frequent family meals on children’s more optimal adiposity indices.

While we expect intervention effects across the sample, we will examine family functioning (e.g., family roles, communication, organization) as a moderator of the effects of the final, bundled intervention [33]. Families’ abilities to plan and implement family meals likely are indicative of overall family functioning [34–36]. Lower functioning families may particularly benefit from the bundled intervention. Also, given that participating in family meals is associated with parents’ [23, 25, 37] and children’s [22] perceptions of family togetherness and that family time around eating is important across socioeconomic groups [34, 37], we will examine parents’ perceptions of the mealtime climate during the Confirming Phase. We expect that engaging in more family meals will contribute to a greater sense of togetherness and mealtime enjoyment [38]. Increased togetherness may contribute to the child’s consumption of healthy foods [39, 40]. In the Confirming Phase, we expect that enhanced mealtime climate will partially mediate the effects of family meal frequency on children’s dietary quality. We are distinguishing family functioning, reflecting systems-level organization that tends to be stable [41], from family mealtime climate, which we expect to be more flexible and influenced by the intervention and frequency of family meals.

## Methods/Design

### Design in the screening phase

In the current Screening Phase, SD intervention components are implemented weekly for 8 weeks, with the exception of the delivery of cookware which occurs once at the beginning of the intervention period. The Screening Phase consists of five, 8-week cycles, each involving different families. Data are collected pre-intervention and immediately post-intervention in the home, with data collectors blind to intervention assignment status. We also assess family meal frequency midway through the intervention to provide additional information on meal frequency over the course of the intervention.

For the SD study, the MOST factorial design includes 6 intervention components with a Usual Head Start Exposure condition; thus, individual participants are randomized to one of 64 experimental conditions. The 64 experimental conditions result from the crossing of 6 SD intervention components, each of which has 2 conditions (present vs. not), and reflect all possible pairings of the intervention components, including a no-intervention condition which serves as the Usual Head Start Exposure condition. This design maximizes the statistical power to provide main effect estimates for each of the 6 intervention components as well as estimates for interactions between the components. It is important to note that in interpreting the results of a trial of this design, it may not be necessary or desirable to base decisions from the Screening Phase purely on statistical significance, which would require controlling for the experiment-wise error rate [42, 43]. Specifically, the focus is on effect sizes and cost. Some SD components are quite costly (i.e., Meal Delivery). In order to justify including them in the bundled intervention, we will require them to have a sizeable effect size relative to less costly components. Other intervention components are less costly (e.g., provision of Cookware/flatware) but may have an intensifying, interactive effect with another intervention component (e.g., home delivery of meal ingredients for cooking). Since interactions are more difficult to detect and typically have smaller effect sizes, if the interactive effect of provision of cookware and flatware with provision of meal ingredients has an effect size large enough to justify the added cost, we will consider including it in the bundled intervention, even if the interaction term in the factorial design in the Screening Phase does not achieve statistical significance.

### Design in the confirming phase

In the Confirming Phase, participants will be randomly assigned to the bundled SD intervention identified in the Screening Phase or to a control group reflecting Usual Head Start (HS) Exposure. Participants in the intervention and control groups will be matched on demographic characteristics to minimize differences not due to the intervention. Data will be collected pre-intervention in the fall of the school year, mid intervention in early spring of the school year and post-intervention in late spring of the school year. Data collectors will be blind to intervention group status.

### Participants and recruitment

Participants are recruited from two HS programs in mixed rural and urban areas of Michigan. HS is a federally funded preschool program serving children ages three to five years and their low-income families; 90% of HS families have annual incomes below the federal poverty guidelines. Collectively, our cooperating HS programs serve more than 1800 preschool-aged children annually; 52% Caucasian; 22% African American; 26% other or biracial; 13% Hispanic. The prevalence of overweight (BMI ≥ 85^th^ percentile and < 95^th^, 16.2%) and obesity (BMI ≥ 95percentile, 17.0%) within these programs is similar to other HS cohorts nationally [3, 44]. SD enrollment exclusions include serious medical problems that affect appetite or eating, significant developmental disabilities that would preclude participation, family anticipating leaving the HS program during the school year, foster care placement or parents who do not speak or understand English. Families are recruited for study participation at HS parent open houses, and via flyers in children’s backpacks and flyers distributed by HS teachers during home visits with families or other family meetings. Enrollment procedures also screen for food allergies. Parents are compensated $10 for returning an initial enrollment packet. After receipt of these packets, research staff contact families by phone to fully describe the study and ascertain eligibility for study participation. After establishing family eligibility and desire to participate in the study, data collectors complete home visits in which written informed consent is obtained followed by administration of pre-intervention assessments. Families are compensated $150 for pre, mid and post intervention assessments during the Screening Phase and we expect to compensate families $200 for the Confirming Phase over a school year. Children’s teachers are also invited to complete behavioral ratings of children and written consent is obtained prior to data collection.

### Intervention descriptions

Table 1 describes the 6 intervention components from most to least intensity of support provided. Participants in the first 5 intervention components receive healthy recipes with detailed instructions addressing preparation techniques. Intervention components 3–5 (Community Kitchen-CK, Didactic Healthy Eating Classes-POPS, and Cooking Demonstrations-CD) are conducted in group settings incorporating a social support element. CK, POPS and CD are implemented by community-based University Extension educators with training in health and nutrition including a registered dietician. Recipes used in the intervention components were developed via a multi-tiered process described below.

**Table 1:** Description of Simply Dinner components and research support for the inclusion of the components

Intervention Component	Receipt of Food to Use at Home	Description	Research Support
1. Meal Delivery (MD)	Yes	Prepared meal (lean protein, vegetable, fruit, whole grain) are delivered weekly to the home ready to heat and eat. Recipes provided.	Meal delivery may be especially important for low-income parents who lack cooking self-efficacy, skills and knowledge [65] and/or who are food insecure and cannot afford healthy foods, although meal delivery has primarily been tested only with elder adult populations [66].
2. Ingredient Delivery (ID)	Yes	Ingredients to make a meal at home (lean protein, vegetable, fruit, whole grain) are delivered weekly to the home. Recipes provided.	Cooperative Extension Systems across the country have begun to supply nutrition education participants with ingredients for recipes prepared during class with the intention that the participants will replicate the recipes at home [67, 68].
3. Community Kitchen (CK)	Yes	Participants attend a group session weekly to make meals (to be cooked at home) from ingredients with support from Extension educators. Recipes provided.	Hands-on cooking experiences have been linked to greater perceptions of self-efficacy in cooking at home [68–70].
4. Didactic Healthy Eating Classes (POPS)	No	Participants attend a weekly group Preschool Obesity Prevention Series (POPS), developed in our prior work [62, 71] based on recommendations from the American Academy of Pediatrics [72, 73], is utilized. The class focuses on increasing intake and variety of fruits and vegetables and reduction of sugar-sweetened beverages. Lessons also address portion sizes and meal planning. Participants will make and taste a dish. Recipes provided. Educational enhancements (e.g., water bottle, children’s book about picky eating) provided.	Since 1969 Cooperative Extension systems across the nation have provided hands-on, interactive nutrition education lessons to low-income parents and other adult caregivers of young children. Today these learner-centered classes are often funded by the Expanded Food and Nutrition Education Program, the Supplemental Nutrition Assistance Program Education, and other granting organizations. Didactic discussions are a core element of the sessions and are often combined with hands-on activities, food demonstrations and taste-testing. They have been found to be effective in improving dietary quality, food cost savings, and food safety for families [74, 75].
5. Cooking Demonstrations (CD)	No	Participants attend a weekly group to watch, listen and taste as the “chef” (Extension educator) describes and makes a main dish. Educational enhancements (e.g., spices) are provided. Recipes are provided.	Many nutrition education programs across the country have begun to integrate healthy food demonstrations by “chefs” into their nutrition education classes. Initial studies of some curricula that combine nutrition education with chef-led food demonstrations have shown positive changes in dietary quality [76], but research has not yet determined if the “chef” element is a major contributing factor to behavioral change in dietary quality and the preparation of healthy meals. Extension educators have been shown to be effective in scaffolding food preparation skills in parents [67]. [77]
6. Cookware/ Flatware Provision	No	Participants receive a new set of matching pans, measuring cups, and a new set of dishes and flatware for use in making and serving meals.	Research on the adequacy of low-income families’ cooking materials is mixed, with some studies showing adequate cookware in the home [78]. However, other studies find not only that low-income families lack basic cooking supplies [79] but that economic shifts have meant that they are less likely to spend limited funds on cookware and flatware [80]. Not having the necessary equipment to prepare meals at home could be a deterrent to healthy eating [79].

#### Selecting Recipes

Recipes for the intervention arms were selected using a multi-step process. First, preliminary recipes were selected from the USDA (e.g., USDA What’s Cooking- USDA Mixing Bowl at https://www.whatscooking.fns.usda.gov/) based on having five or fewer ingredients and cooking times of 30 min or less. Recipes using nut products were excluded. Next, preliminary recipes were reviewed by partnering HS agencies to rate the “face validity” with regard to which recipes would be attractive to families. Recipes were discarded at the recommendation of the community partners. In the third phase of the selection process, preliminary recipes were rated for nutrition quality using the Healthy Meal Index. [45] The index provides a score for dietary adequacy, ranging from 0 – 65, reflecting the inclusion of foods in the meal that are recommended for a healthy diet, such as fruits, vegetables, lean proteins, lean dairy and whole grains. A moderation score was calculated, ranging from 0 – 40, which reflects the absence of foods to be used in moderation, such as butter and foods with added sugar. Scores were summed to reflect a total HMI score, ranging from 0 – 106, with higher values indicating healthier meals. Recipes with HMI total scores of 90 or greater were selected for use in the interventions. Different recipes were assigned to each intervention component that utilizes recipes (MD, ID, CK, POPS, and CD).

### Data collection procedures

Data collectors, who are blind to family intervention status and not involved in the implementation of intervention activities, were trained by the study directors in all protocols. Prior to the start of the intervention (pre) and following the conclusion of the intervention (post), data collectors complete home visits to measure child height and weight and child triceps skinfold and to administer questionnaires using computer-assisted administration. Maternal height and weight are assessed and will be treated as a covariate (described below). If the child is not available during the home visits, child height and weight are measured by data collectors at children’s HS programs. Teachers of participating children complete questionnaires on child behavior at pre and post assessments. Midway through the intervention, data collectors conduct brief questionnaires with parents over the telephone to ascertain current frequency of family meals at home. Data quality is assessed on an ongoing basis.

### Primary outcome measures-screening phase

Alphas noted in the measures below are those reported by the developers of the measures as data collection in the current study is ongoing.

#### Family meal frequency

Family meal frequency is assessed in three ways using items adapted from Storfer-Isser and Musher-Eizeman [46, 47] describing how many nights out of 7: a) the family ate a meal together at home in the same place at the same time; b) the family consumed fast food meals, such as hamburgers and french fries; and, c) the meal was prepared “from scratch”, ready-made foods such as cooked chicken from a grocery store, or was prepared from a combination of “from scratch” and ready-made foods. The “family” is operationalized as those family members usually present for meals. The rationale for these items is that cooking from scratch and/or using some ready-made foods usually reflects healthier meals than eating out, eating carry out food or fast food [48]. We will calculate a mean score for each of these three variables, reverse scoring frequency of fast food meal consumption such that higher scores mean healthier family meals. We will also create a composite mean family meal frequency variable reflecting the three outcomes, scored such that higher scores indicate greater family meal frequency.

#### Children’s dietary quality

Children’s dietary intake is assessed using the Block Dietary Data Systems Kids Food Screener—Last Week (Version 2) [49]. This screener is designed to assess children’s dietary intake by food groups and asks about food eaten during the last week with questions about individual portion sizes. To assess diet quality, we focus on the combined food group intake of fruits (including fruit juices) and vegetables (excluding potatoes and french fries) with outcomes measured in average daily servings. Fruit and vegetable consumption is generally associated with overall diet quality but intervening to increase fruit and vegetable consumption in preadolescents has recently been shown to improve diet quality but not decrease caloric consumption [50]. Accordingly, our focus also includes average number of servings of sugar- sweetened beverage intake which can contribute substantially to total caloric consumption [51]. Although more detailed dietary intake data can theoretically be obtained by administration of multiple 24-h dietary recalls ascertained by maternal/caregiver report, we chose a Food Screener as our primary measure of dietary intake because the immense participant and interviewer burden necessitated by multiple 24-h dietary recalls is not justified in this context. The Food Screener has good relative validity and assesses food intake over a longer period, suggesting the ability to capture food intake that may be missed in 3 days of 24-h dietary recalls [49].

### Primary outcome measures-confirming phase

#### Anthropometry

The SECA stadiometer and the Detecto DR-550-C are used to measure height and weight, respectively. For triceps skinfold, a measure of body fatness, a Gulick II measuring tape and Lange Calipers are used. Mothers and children are weighed without shoes or heavy clothing. Body mass index (BMI), used as an indicator of obesity prevalence, will be calculated and child BMI z-scores will be derived using US Centers for Disease Control and Prevention reference growth charts for age and sex. [52]

### Secondary outcome measures- screening and confirming phases

#### Cooking self-efficacy

Maternal self-efficacy is assessed via the Cooking Techniques and Meal Preparation Self-Efficacy Scale [46]. This 5-item scale (α = .90) reports on a variety of self-efficacy domains including using recipes, cooking from basic healthy ingredients, using a variety of fruits and vegetables, and cooking with limited time and money. A total mean cooking self-efficacy score will be calculated.

#### Barriers to family meals

Barriers to meal preparation [46, 47] (α = .82) is assessed via 18 items as negative perceptions of time and effort barriers to planning and preparing meals, negative perceptions of acceptability of the meal to the child and family, and preferences for other activities besides cooking. A mean score will be calculated.

#### Mealtime resources

We created a 35-item inventory of the presence of basic cooking utensils (e.g., measure cups), eating utensils (e.g., plates, silverware), cookware (e.g., pan, skillet), appliances (e.g., working oven), and other (e.g., table and chairs) to be used in the current study to assess the sum and mean number of available resources for the preparation and service of family meals.

#### Family mealtime climate

Priority for family meals (e.g., family members are expected to be home for dinner; α = .82), atmosphere and enjoyment of family meals (e.g., eating brings people together in an enjoyable way; α = .73), and rules at family meal (e.g., manners are important at the dinner table; α = .60) are assessed using the 14-item Family Eating Attitudes and Behavior Scale [53]. The Mealtime Interaction Coding System (MICS) (Dickstein S, Hayden L, Schiller M, Seifer R, San Antonio W: Providence Family Study mealtime family interaction coding system, unpublished) will be used to assess mealtime climate via mealtime interactions in the Confirming Phase. We are particularly interested in aspects of the mealtime climate including mealtime communication, affective interaction, and interpersonal involvement at meals that are coded with the MICS. Mean scores will be calculated.

### Moderators

#### Family functioning

The MacMaster Family Assessment Device [54] is used to assess family problem solving (α = .74), communication (α = .75), roles (α = .72), affective responsiveness (α = .83), affective involvement (α = .78), behavior control (α = .72), and general functioning (.92) yielding subscale and mean family functioning scores.

### Covariates

#### Parental psychosocial functioning

Parental psychosocial functioning is assessed as mean depressive symptoms reported via the Center for Epidemiological Studies [55] (α = .88 in a sample of low-income mothers of preschoolers [56]).

#### Child behavior

HS teachers rate children’s internalizing and externalizing behavior problems using the Child Behavior Checklist [57, 58], to account for child behavior that could impact family meals (α = .89 and .92 for the internalizing and externalizing subscales).

#### Demographics

Food security, using the USDA Household Food Security Scale [59], family income, parental age, education, child age, sex, and parent and child ethnicity and race are collected at enrollment into the study.

### Process evaluation

To assess fidelity to the protocol by educators, CK, CD, and POPS are observed by research staff. Educators complete self-reports regarding any barriers to implementation and the degree to which activities are implemented to completion. As part of the process evaluation, the cost of implementation of each SD intervention component, per participant, will be calculated at the conclusion of the Screening Phase. Families are contacted each week of the intervention by text or phone to ascertain their satisfaction with the SD activities to which they are assigned and to inquire as to whether the provided foods were consumed, and what, if any, additional foods or beverages were consumed with the SD foods.

### Data analysis and power calculations

#### Data analysis: screening phase

The primary outcomes for the Screening Phase are the frequency of family meals in the past week and children’s dietary quality over the past week. As described previously, we will first test three family meal frequency outcomes describing how many nights out of 7 the family: a) ate a meal together at home in the same place at the same time; b) consumed fast food meals; and, c) prepared the meal “from scratch”, ready-made foods such as cooked chicken from a grocery store, or was prepared from a combination of “from scratch” and ready-made foods. Children’s dietary quality is operationalized as the average daily servings over the past week of: a) fruits and vegetables and b) sugar sweetened beverages. We will test each meal frequency outcome and each child dietary quality outcome in separate models initially. As warranted, we will use a composite meal frequency variable and a composite dietary quality score, reverse scored as appropriate such that higher scores reflected greater family meal frequency and more optimal dietary quality. Secondary outcomes will include mean perceptions of self-efficacy, barriers and mealtime resources.

The effects of the 6 intervention components on these outcomes will be examined by means of a factorial experiment involving the following factors: 1) Meal Delivery (provided vs. not); 2) Ingredient Delivery (provided vs. not); 3) Community Kitchen (provided vs. not); 4) POPS healthy eating classes (provided vs. not); (5) Cooking Demonstrations (provided vs. not); 6) Flatware/Cookware (provided vs. not); and, will also include a no-intervention, Usual Head Start exposure condition. Initially we will use repeated measure ANOVA models to test whether each factor has a significant effect on frequency of family meals and dietary quality over an 8 week-period pre- to post-intervention. For each of the intervention components, we will determine whether there is a difference in change across each 8 week-period (i.e., post-intervention vs. pre-intervention), indicating whether there is a main effect of each component on the change pre-post difference. We will also include two- and three-way interactions between components to identify the effects of interactions between intervention components on outcomes. Next, we will make decisions about intervention components to be included in the Confirming Phase, based on Screening Phase analytic results. We will use a modified version of a decision making approach frequently used in engineering [60], which emphasizes main effects, using interactions as additional valuable information. This emphasis is consistent with our objective of identifying a final set of bundled components in which each component is making a detectable contribution to the overall effect, and any inactive components have been eliminated.

#### Sample size and power calculations: screening phase

The sample size calculation is done using the Factorial Power Plan SAS Macro [61]. We assume an attrition rate of 5% based on prior experience working with HS (e.g. [62],). Based on these assumptions, with 80% power under a two-sided Type I error alpha = 0.05, a sample of 512 research participants, or 8 participants for each of the 64 cells reflecting each combination of intervention components or the Usual Head Start Exposure, will allow us to detect a main effect or interaction effect size of d = .25 or greater or a standardized regression coefficient b = 0.13.

#### Data analysis: confirming phase

The primary objective in the Confirming Phase is to determine whether the bundled intervention vs. Usual Head Start Exposure is more effective in reducing children’s adiposity indices. The primary outcomes will be BMI z-score and triceps skinfold thickness. Secondary outcomes will be perceptions of self-efficacy, barriers to meals, and resources for meals, the frequency of family meals, and children’s dietary quality. Baseline comparability of the two groups will be assessed using t-test for continuous variables and χ^2^ tests for categorical variables. To account for clustering of children within a HS classroom, Proc Mixed in SAS with a random intercept for the classroom will be used. We will employ logistic and Poisson regression models using General Estimating Equations techniques [63] to account for classroom clustering to compare changes in the prevalence of obesity and overweight across the 2 groups. Also, to assess consistency of the intervention effects over subgroups, we will perform subgroup analysis separately for boys and girls, Caucasian and non-Caucasian children, and children who are overweight and non-overweight at baseline. All analysis will be based on intention-to-treat principle.

In addition, we will test the study’s conceptual model (Fig. 1), which illustrates hypothesized mediated and moderated relationships, in the Confirming Phase. We will test several mediation pathways: first, whether increased self-efficacy, decreased barriers and increased resources mediate the effects of the intervention on family mealtime frequency; and, second, whether increased frequency of family meals and improvements in dietary quality mediate intervention effects on improvements in children’s adiposity indices. We will also examine improvements in family mealtime climate as mediating the effects of increased frequency of family meals on children’s dietary quality. Mixed models and GEE will be used for analyses that test mediation. Tests for mediation effects will be performed following MacKinnon et al. [64] Finally, we will test family functioning as a moderator of intervention effects by including in the model an interaction term between the family functioning variable and the intervention indicator.

#### Sample size calculations and power: confirming phase

A sample of 250 (n = 125 participants per group) will be recruited. Assuming attrition of 20% we will achieve a final sample size of 100 participants per group (n = 200 minimum). This sample size will enable detection of small to moderate effect sizes of d = .40 for continuous outcomes with a power of 80%, α = .05. For binary outcomes, we will be able to detect a 38% relative risk reduction with a power of 80%, α = .05.

## Discussion

The SD study will provide important information about the types of supports for family meals most robustly associated with the frequency of family meals at home, improvements in children’s dietary quality, and, importantly, children’s more optimal adiposity indices. If the most intense intervention component (delivering pre-made healthy family meals weekly to the home) does not contribute to healthy outcomes, this result would provide important evidence that less intense interventions are unlikely to be effective and may not be worth further pursuing. Alternatively, if we discover that provision of cookware and flatware alone significantly increases healthy outcomes, this result would provide evidence that a simple redirection of resources to this concrete provision of meal-related materials may be a worthwhile investment. If we demonstrate that didactic classes have little impact, or are only effective in the context of providing concrete material resources (in the form of food or equipment), policy makers might consider how to allocate limited resources for the greatest benefit. Thus, the SD study will result in the determination of the types of interventions necessary to uncover or intensify the effects of traditional interventions focused on nutrition knowledge and cooking skills. We expect that the study will have significant implications for policy makers seeking to improve dietary quality and prevent childhood obesity through consideration of how to allocate limited resources for the greatest benefit.

## Abbreviations

BMI: Body mass index
CD: Cooking demonstration
CK: Community kitchen
CW: Cookware delivery
HS: Head start
ID: Ingredient delivery
MD: Meal delivery
NIFA/AFRI: National institute of food and agriculture/agriculture and food research initiative
POPS: Preventing obesity in preschoolers series
RCT: Randomized controlled trial
SD: Simply dinner study
USDA: United States department of agriculture
